# Research trends of mesenchymal stem cells application in orthopedics: A bibliometric analysis of the past 2 decades

**DOI:** 10.3389/fpubh.2022.1021818

**Published:** 2022-09-26

**Authors:** Zhibo Deng, Fenqi Luo, Yuan Lin, Jun Luo, Dianshan Ke, Chao Song, Jie Xu

**Affiliations:** Department of Orthopedics, Fujian Clinical Research Center for Spinal Nerve and Joint Diseases, Shengli Clinical Medical College of Fujian Medical University, Fujian Provincial Hospital, Fuzhou, China

**Keywords:** mesenchymal stem cell, orthopedics, bibliometric analysis, hotspot, Web of Science

## Abstract

**Background:**

Bibliometric analysis and visualization tools were used to determine the development trend of mesenchymal stem cells (MSCs) in orthopedics in the past 20 years, so as to guide researchers to explore new directions and hotspots in the field in the future.

**Methods:**

In the Web of Science Core Collection, all articles about the application of MSCs in orthopedics from 2002 to 2021 were searched. The qualitative and quantitative analysis was performed based on Web of Science and CiteSpace software.

**Results:**

A total of 2,207 articles were retrieved. After excluding non-article articles such as review and letter and non-English language articles, 1,489 articles were finally included. Over the past 2 decades, the number of publications on the application of MSCs in orthopedic diseases increased. Among them, the United States, China, Japan and the United Kingdom have made significant contributions in this field. The most productive institution was Shanghai Jiao Tong University. *Journal of Orthopedic Research* published the largest number of publications. The journal with the highest citation frequency was *Experimental Hematology*. The authors with the highest output and the highest citation frequency on average were Rochy S. Tuan and Scott A. Rodeo, respectively. “Mesenchymal stem cell”, “*in vitro*” and “Differentiation” were the top three keywords that appeared. From the keyword analysis, the current research trend indicates that the primary research hotspots of MSCs in orthopedics are the source of MSCs, *in vitro* experiments and the differentiation of MSCs into bone and cartilage. The frontiers of this field are the combination of MSCs and platelet-rich plasma (PRP), the treatment of knee diseases such as osteoarthritis, osteogenic differentiation, and the application of biological scaffolds combined with MSCs.

**Conclusion:**

Over the past 2 decades, the application of MSCs in orthopedic diseases has received increasing attention. Our bibliometric analysis results provide valuable information and research trends for researchers in the field to understand the basic knowledge of the field, identify current research hotspots, potential collaborators, and future research frontiers.

## Introduction

Mesenchymal stromal/stem cells (MSCs) are non-hematopoietic adult stem cells derived from mesoderm (1). It can be found in bone marrow, fat, synovium, tonsil, peripheral blood, amniotic fluid, umbilical cord blood and other tissues (2). It has the potential of self-replication, self-division, self-renewal, and multidirectional differentiation, to repair tissues and maintain their homeostasis (3, 4). In addition to the powerful immunomodulatory properties to regulate neighboring cells and enhance tissue repair ability, MSCs can also differentiate into resident cells to replace damaged tissues (5). Therefore, it plays a key role in tissue healing and regenerative medicine.

At present, MSCs have been proved to be able to treat a variety of diseases, such as hematological diseases, cardiovascular system diseases, digestive system diseases, nervous system diseases, autoimmune diseases and other diseases (6). In addition, MSCs have been proved to have the potential ability to differentiate into bone, cartilage, tendon, ligament, muscle, fat and other cells (7). Meanwhile, MSCs have good immunomodulatory activity and paracrine ability (8), which can synthesize and release cytokines, stimulate progenitor cell proliferation, inhibit cell apoptosis, and promote tissue repair. They are the seed cells with the most promising clinical application in regenerative medicine (9), and have been widely used in the treatment of orthopedic diseases (10), especially for sports injury diseases, such as cartilage injury, meniscus injury, cruciate ligament injury, achilles tendon rupture, rotator cuff injury, etc (11). Bibliometrics is a tool to evaluate published articles through mathematical and statistical methods, and bibliometrics analysis has been widely used in various medical disciplines. It enables beginners to intuitively, systematically and comprehensively understand the development process and hot trends of a particular field (12). CiteSpace is a software that visualizes information to present and identify new trends. The software can display the structural relations and evolution rules of a research field for beginners from different dimensions and levels in the way of knowledge map (13). At the same time, the software is easy to obtain, easy to operate and readable, so it has been widely used in bibliometrics analysis and other fields.

At present, the research in this field of MSCs in orthopedic diseases mainly focuses on the mechanism research and clinical observation, but there is a lack of clear combing in the hotspot trend and intuitive structure display. This study intends to analyze the application of MSCs in orthopedic diseases in the past 20 years through bibliometrics analysis. The authors, publishing institutions, keywords and other elements are presented in the form of knowledge map, so as to comprehensively understand the research background of MSCs in the treatment of orthopedic diseases, predict the future development trend and hotspots, and provide reference for further related research.

## Methods

### Data collection and search strategy

Considering that Web of Science (WoS) is an important academic database in the world and is often used in bibliometric analysis (14, 15), we chose the WoS core collection database as the literature source. The time period of the search was 2002–2021. The search strategies were as follows: TS = (mesenchymal stem cell^*^ OR MSC^*^ OR bone marrow stromal stem cell^*^ OR mesenchymal stromal cell^*^ OR bone marrow stromal cell^*^ OR mesenchymal progenitor cell^*^) AND TS= (orthopedic^*^ OR orthopedics department OR Clinical orthopedic^*^ OR Bone science OR orthopedic^*^). A total of 2,207 studies were retrieved, and only 1,521 articles were exported for records and cited references in the format of plain text files. All data used in this work were downloaded from public databases, therefore did not require ethics committee approval or informed consent. Detailed retrieval strategy is listed in Table 1.

**Table 1:** Data sources and flow of retrieval strategy.

**Content**			
Data sources	Web of Science Core Collection		
Publication date	1 January 2002–31 December 2021		
Languages	English only		
Document type	Article only		
Search strategy			
	#1	139,289	TS = (mesenchymal stem cell* OR MSC* OR bone marrow stromal stem cell* OR mesenchymal stromal cell* OR bone marrow stromal cell* OR mesenchymal progenitor cell*)
	#2	44,985	TS = (orthopedic* OR orthopedics department OR Clinical orthopedic* OR Bone science OR orthopedic*)
	#3	2,207	#1 AND #2
	#4		Document types: (Article)
	#5		Language: (English)
	#6	1,521	#1 AND #2 AND #3 AND #4 AND #5

### Statistical analysis

Information including annual publications and journal distributions was obtained from the WoS database. The chart of annual publications was generated using Microsoft Excel 2019. Then, the data was imported into CiteSpace (5.8R3), and 1,489 records were retained after removing duplications. “Time Slicing” was set for one per slice from 2002 to 2021. All the options in “Term Source,” “pathfinder,” “pruning sliced networks,” and “Pruning the merged network” in pruning were selected. Country, institutions, journal and keywords in node types were selected to perform co-occurrence analysis, respectively. Besides, the node “Keyword” was used for cluster analysis. In the co-occurrence maps, the size of nodes represents the degree of co-occurrence or the frequency of citation, and the connection between nodes represents the cooperation and co-occurrence relationship. It is worth noting that nodes with high frequency and high centrality are the critical points in the field, and therefore these are also the core of our study (13).

## Results

### Annual publication outputs and trends

**Figure 1:**
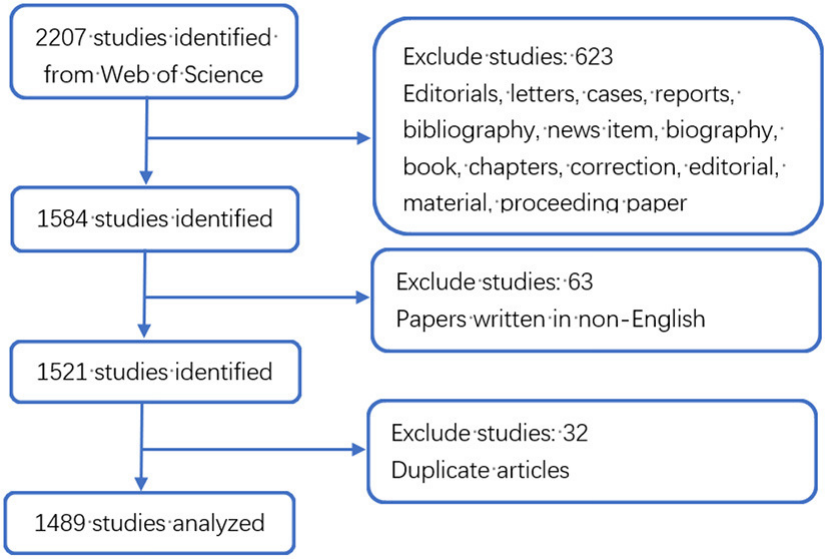
Detailed flow chart of search and screening.

**Figure 2:**
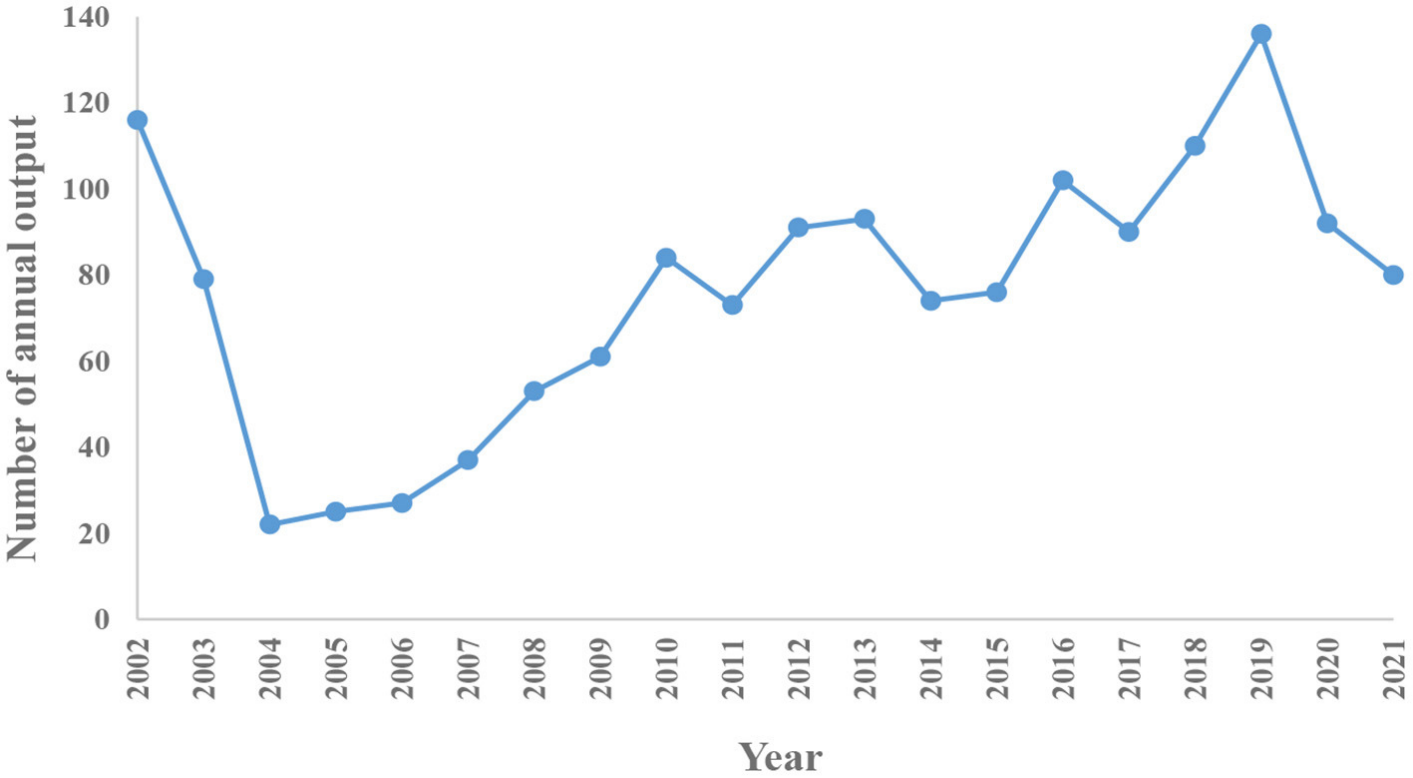
The annual number of publications and trends on MSCs in orthopedics between 2002 and 2021.

As shown in Figure 1, a total of 2,207 literatures were retrieved. We only included articles and English language documents, and finally the remaining 1,521 publications retained a total of 1,489 articles after excluding duplicate articles. It is known that the research in the field of MSCs in orthopedic diseases mainly focuses on basic scientific researches and clinical researches. As shown in Figure 2, according to the annual distribution of publications, with year as the abscissa and number of publications as the ordinate, the continuous exploration of MSCs in orthopedics is visually presented. It can be seen that the number of publications in 2004 was the lowest, with 22. The highest was 136 in 2019. The research period can be divided into two stages: from 2002 to 2004, the number of annual publications showed a downward trend, and from 2004 to 2019, the number of annual publications showed a gradual upward trend. Since 2019, there has been a slow decline in the research results of MSCs in orthopedic diseases worldwide, which may be related to the COVID-19 pandemic, as it leads to a decline in scientific research productivity. In 2002, the reason for the sudden increase in publications was considered a breakthrough in stem cell basic research. Since 2010, there have been more than 70 articles published annually, indicating that MSCs have always been a research hotspot in the treatment of orthopedic diseases, and a stable research environment has been formed.

To better study the dynamics change of MSCs in orthopedics over the past 20 years, we divided the period into two periods based on the 10-year period. The two periods were the 2002–2011 period and 2012–2021 period, as shown in Table 2. there were 577 publications in the first period (2002–2011), while there were 912 publications in the second period (2012–2021), an increase of 158%. This increasing trend is also reflected in the other variables considered in the table. In terms of authors, the first period involved 2,596 scholars, while the second period had a total of 4,847 authors. This means that with the development of MSCs in the field of orthopedics, increasing scholars began to pay attention to this field and published relevant research results. As for journals, the number of journals increased from 169 in the first period to 275 in the second period, an increase of 163%. In terms of countries, there were only 39 countries in the first period and 63 countries in the second period, representing an increase of 162%. This means that about a third of the countries worldwide are involved in MSCs research in the field of orthopedics. It is worth noting that the sample recorded a total of 66,498 citations over the time frame analyzed, for a total average of 44.66 citations per article. The average number of citations per article in the first period was 77.27 times, but only 24.03 times in the second period. Considering that the publications of the second period were published recently, the cumulative number of citations was still small. Whether there is a lack of representative articles in the second period, it still needs a certain amount of time to accumulate the citations and then analyze again.

**Table 2:** Comparison of the major characteristics of the two periods (2002–2011 and 2012–2021).

**Period**	**Articles**	**Authors**	**Journals**	**Countries**	**Citations**	**Average citations**
2002–2011	577	2,596	169	39	44,583	77.27
2012–2021	912	4,847	275	63	21,915	24.03
**Total**	1,489	675	369	103	66,498	44.66

### Analysis of countries

**Figure 3:**
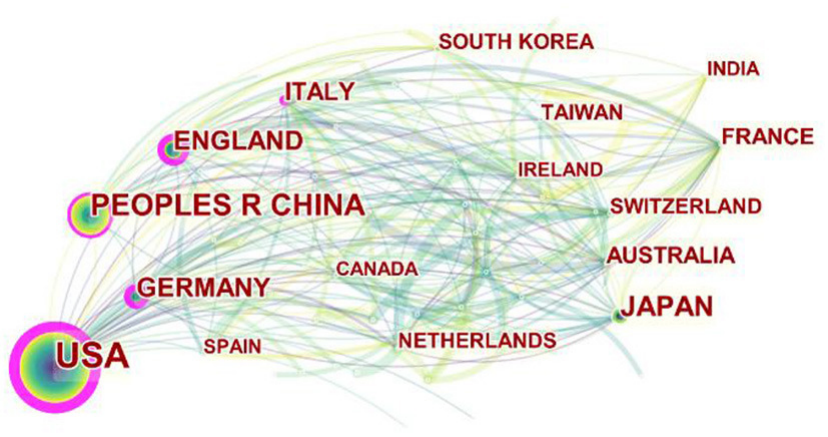
The cooperation network map of countries related to MSCs in orthopedics from 2002–2021. Node size represents the number of output. The thicker the pink outer ring of the node, the higher the centrality.

The 1,489 articles came from 65 different countries or regions. Among them, The United States (USA) (553) was the most productive country, China (230) was the second, and Japan (134) was the third. England (120) was the fourth and Germany (109) was the fifth. The top 5 countries with total citations were USA (33,025), England (6,384), Japan (6,209), Italy (5,953) and China (5,515). As can be shown in Figure 3, although USA had the largest number of publications, its publications had gradually declined in recent years. Publications of other countries, such as England and Germany, increased with each year. Although the Netherlands was not in the top 5 in terms of the number of publications and the total number of citations, the average number of citations was the highest. For the average citation times of each article, the top 5 countries were Netherlands (73.15), Italy (67.65), France (62.47), USA (59.72), Switzerland (54.56).

### Analysis of institutions

**Figure 4:**
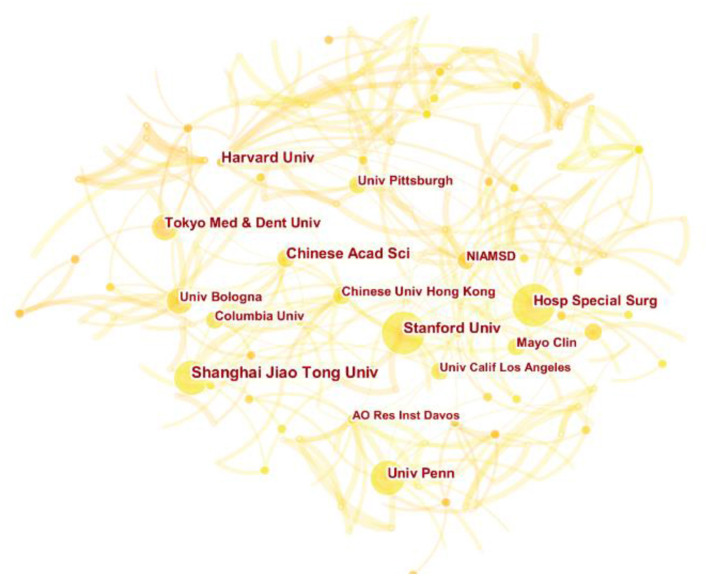
The cooperation network map of institutions related to MSCs in orthopedics from 2002–2021.

A total of 1,711 institutions have participated in the research of MSCs in orthopedics. The top six institutions for the total number of publications were Shanghai Jiao Tong University (33), Harvard University (29), Stanford University (28), Columbia University, University of Pennsylvania (22), Mayo Clinic (22). Except the first one, the other five institutions were located in USA. Figure 4 clearly shows the close and complex collaboration between different institutions, which shows that there is still little inter-institution cooperation. While these institutions were strong in scientific research, the lack of connectivity between nodes indicates weak collaboration between institutions. Stanford University and Hospital for Special Surgery were the most active institutions in the cooperative relationship and the most influential institutions. They were critical institutions devoted to the research of MSCs in orthopedic diseases.

### Analysis of journal

As a medium for the exchange of research results, journals play a vital role in promoting cooperation and the advancement of scientific research. From 2002 to 2021, there were 51 journals devoted to MSCs in orthopedics research. Table 3 lists the top 10 journals with the most publications of MSCs in orthopedics, which are representative journals in this field. As shown in Table 3, *Journal of Orthopedic Research* published the largest number of publications of MSCs in orthopedics with 402 articles, accounting for 26.43% of the total articles, but the average cited frequency was only 46.03. Although *Biomaterials* (47) was ranked the second in terms of the number of publications, the average citation frequency of *Biomaterials* was also ranked the second (158.51). The third-most published Journal was *Journal of Tissue Engineering and Regenerative Medicine* (28). *Experimental Hematology* (25), which ranked fourth in the number of publications, was the journal with the highest citation frequency (174.04). At the same time, the impact factors (IF) of these 10 journals are between 3.084 and 12.479. There are 6 journals with IF < 4.000, 3 journals with IF between 4.000 and 9.000, and 1 journal with IF > 10.000. The IF of *Biomaterials* (12.479) is the highest among the top 10 journals in number of publications.

**Table 3:** Top 10 Journals With publications related to MSCs in orthopedics between 2002 and 2021.

**Rank**	**Journal**	**Publications**	**Total times cited**	**Mean times cited**	**Impact factor (2020)**	**JCR**
1	*Journal of Orthopedic Research*	402	18,503	46.03	3.494	Q1
2	*Biomaterials*	47	7,450	158.51	12.479	Q1
3	*Journal of Tissue Engineering and Regenerative Medicine*	28	645	23.04	3.963	Q2
4	*Experimental Hematology*	25	4,351	174.04	3.084	Q3
5	*American Journal of Sports Medicine*	23	1,273	55.35	6.202	Q1
6	*Bone*	23	2,048	89.04	4.398	Q2
7	*Biochemical and Biophysical Research Communications*	23	1,608	69.91	3.575	Q2
8	*Acta Biomaterialia*	22	849	38.59	8.947	Q1
9	*European Cells & Materials*	18	733	40.72	3.942	Q1
10	*Journal of Materials Science-Materials in Medicine*	18	1,790	99.44	3.896	Q2

### Analysis of authors and cooperative relationship

**Figure 5:**
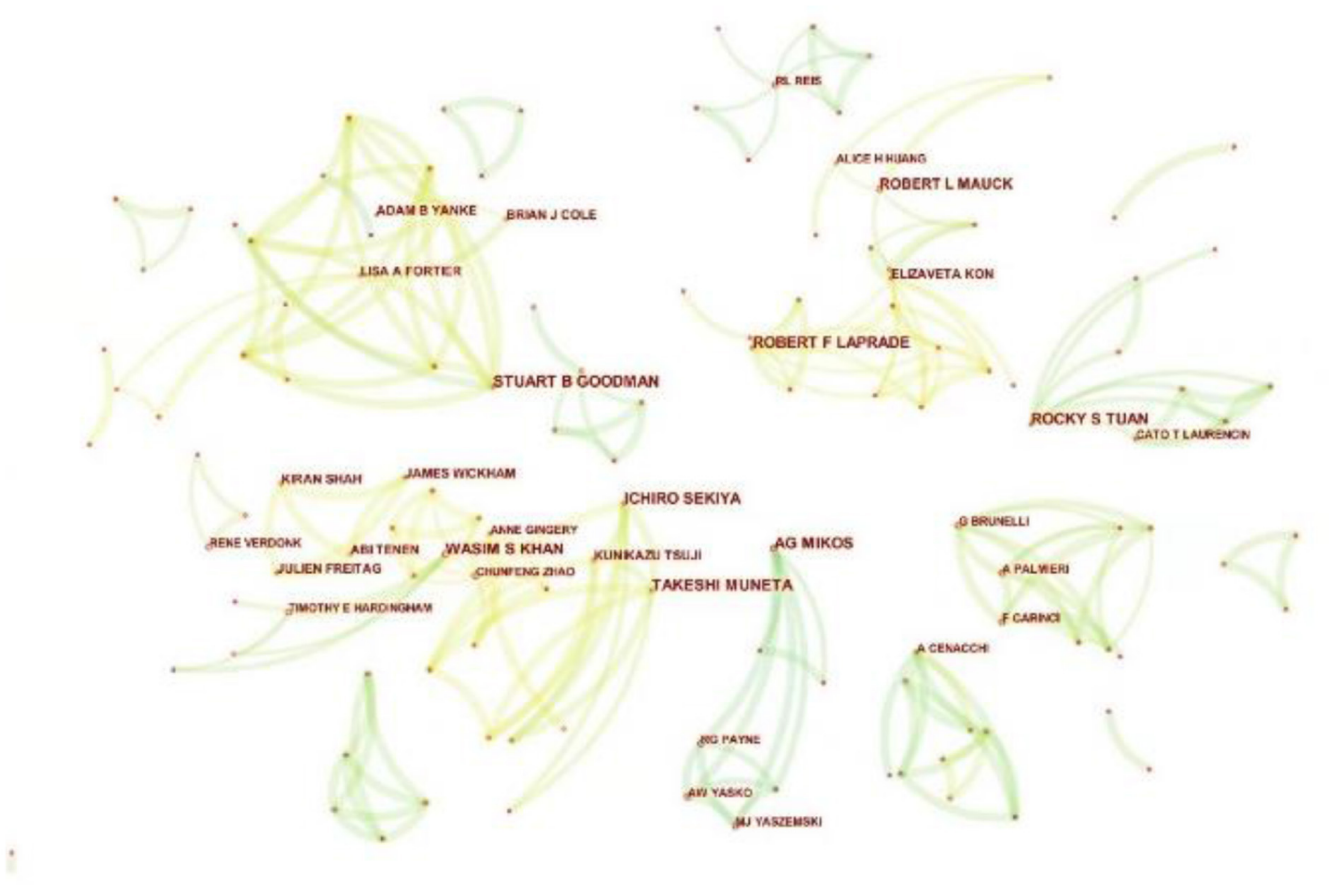
The cooperation network map of productive authors related to MSCs in orthopedics from 2002–2021. Each node indicates an author, and different colors in the nodes indicate different publication years. At the same time, the connecting line between nodes indicates the collaborative relationship between authors.

By analyzing the authors of the included literature, the representative scholars and the main research population in the research field can be grasped. The 1,489 articles included a total of 7,918 authors. Table 4 lists the top 10 authors. The top three authors with the most publications were Rochy S. Tuan (13), Takeshi Muneta (12), and Ichiro Sekiya (12). Meanwhile, in terms of average citation times, the top four authors were Scott A. Rodeo (100.9), Rochy S. Tuan (74.38), Takeshi Muneta (48.42) and Ichiro Sekiya (48.42). Rochy S. Tuan (National Institutes of Health, USA), the most productive author in the field, has been focusing on basic researches such as the multilineage MSCs differentiation potential of human trabecular bone-derived cells since 2002. Figure 5 is the cooperation network map of productive authors. It can be seen that Takeshi Muneta (12) and Ichiro Sekiya (12), who ranked the second and the third in the number of publications, cooperated very closely, and both of them worked in Tokyo Medical and Dental University (Japan). Both scholars devoted to the basic research of meniscal injury and osteoarthritis in knee. But on the whole, there was not much cooperation among the authors in this field, and the authors were not closely related to each other. For the further development of this field, it is suggested to strengthen the cooperation among scholars.

**Table 4:** The top 10 authors in the MSCs for orthopedics ranked by publication number.

**Rank**	**Author**	**No. of publications**	**No. of citations**	**Mean times cited per study**
1	Rochy S. Tuan	13	967	74.38
2	Takeshi Muneta	12	581	48.42
3	Ichiro Sekiya	12	581	48.42
4	Scott A. Rodeo	10	1,009	100.9
5	Stuart B. Goodman	10	343	34.30
6	Tunku Kamarul	10	299	29.90
7	Kunikazu Tsuji	9	330	36.67
8	Peter V. Giannoudis	9	314	34.89
9	Chunfeng. Zhao	9	163	18.11
10	Norbert. Telmon	9	127	14.11

### Analysis of keyword

**Figure 6:**
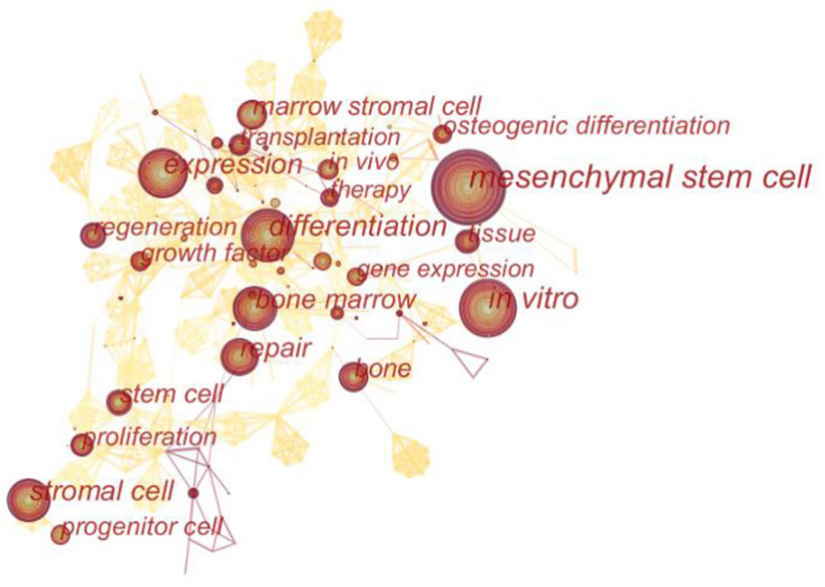
Co-occurrence map of keywords in MSCs in orthopedics from 2002–2021.

**Figure 7:**
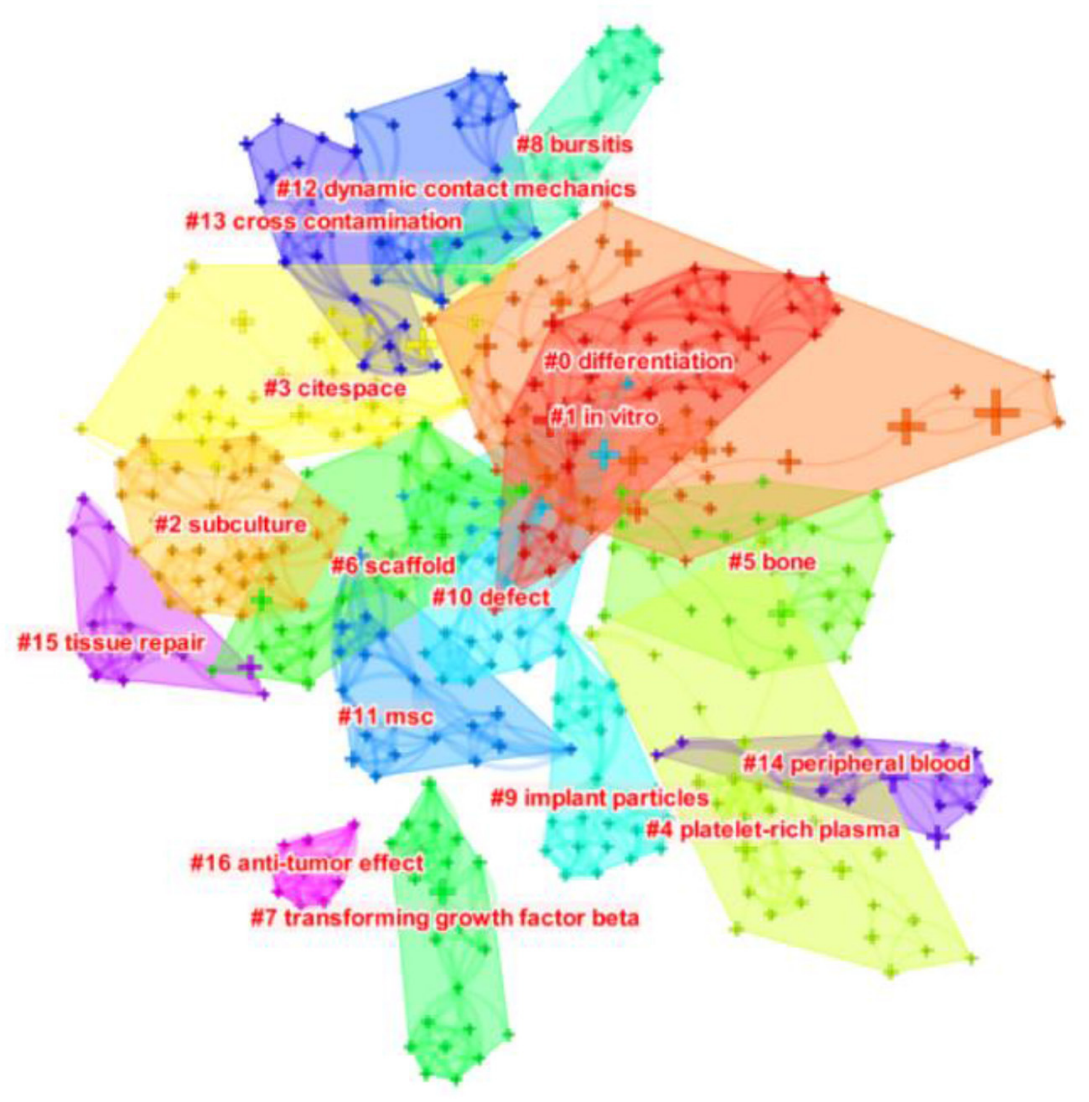
The clustering map of keywords related to MSCs in orthopedics from 2002–2021. The different colored squares in the figure represent different clusters.

Keywords represent the primary topics of published articles (16). Co-occurrence analysis of keywords can help scholars to comprehensively understand the relationship between keywords, and to analyze the relationship between various topics in this field. Further cluster analysis is helpful for thorough analyze the research progress in this field. The lines between nodes in the co-occurrence map of keywords represent their co-occurrence relationship, and the thickness of the lines represents the number of co-occurrence times. As can be seen from Figure 6, the keywords co-occurrence analysis network consists of 457 nodes and 1,537 lines. The high-frequency and high-centrality keywords are listed in Table 5. The top 10 keywords in frequency were “mesenchymal stem cell” (649), “*in vitro*” (304), “Differentiation” (295), “Stem cell” (234), “Bone marrow” (213), “Expression” (211), “Stromal cell” (208), “Repair” (176), “Bone” (136), “Marrow stromal cell” (134). The top 10 keywords in centrality were “Tissue engineering” (0.09), “Collagen” (0.08), “Transplantation” (0.07), “Defect” (0.07), “Marrow” (0.07), “Bone marrow” (0.06), “Cell” (0.06), “Bone morphogenetic protein 2” (0.06), “Injury” (0.06), “Expression” (0.05). Keyword clustering analysis is a process to simplify keywords into a small number of clusters based on the co-occurrence network map (17). The structural features between clusters highlight the important connections of key nodes, which can reflect the research hotspots and trends in the field. As shown in Figure 7, the sequence number was ranked according to the size of the cluster, which divided keywords into different detailed topics. The number of nodes in each cluster was larger than 15, and the silhouette was larger than 0.8, indicating that clusters can be distinguished from each other. Keywords of MSCs in orthopedic disease can be divided into 17 clusters, in order: “differentiation” #0, “*in vitro*” #1, “subculture” #2, “Citespace” #3, “platelet-rich Plasma” #4, “bone” #5, “scaf-fold” #6, “transforming growth factor beta” #7, “bursitis” #8, “implant particles” #9, “defect” #10, “MSC” #11, “dynamic contact mechanics” #12, “cross contamination” #13, “peripheral blood” #14, “tissue repair” #15, “anti-tumor effect” #16.

**Table 5:** The top 10 keywords co-occurrence frequency and centrality ranking.

**Rank**	**Keyword**	**Frequency**	**Rank**	**Keyword**	**Centrality**
1	Mesenchymal stem cell	649	1	Tissue engineering	0.09
2	*in vitro*	304	2	Collagen	0.08
3	Differentiation	295	3	Transplantation	0.07
4	Stem cell	234	4	Defect	0.07
5	Bone marrow	213	5	Marrow	0.07
6	Expression	211	6	Bone marrow	0.06
7	Stromal cell	208	7	Cell	0.06
8	Repair	176	8	Bone morphogenetic protein 2	0.06
9	Bone	136	9	Injury	0.06
10	Marrow stromal cell	134	10	Expression	0.05

**Figure 8:**
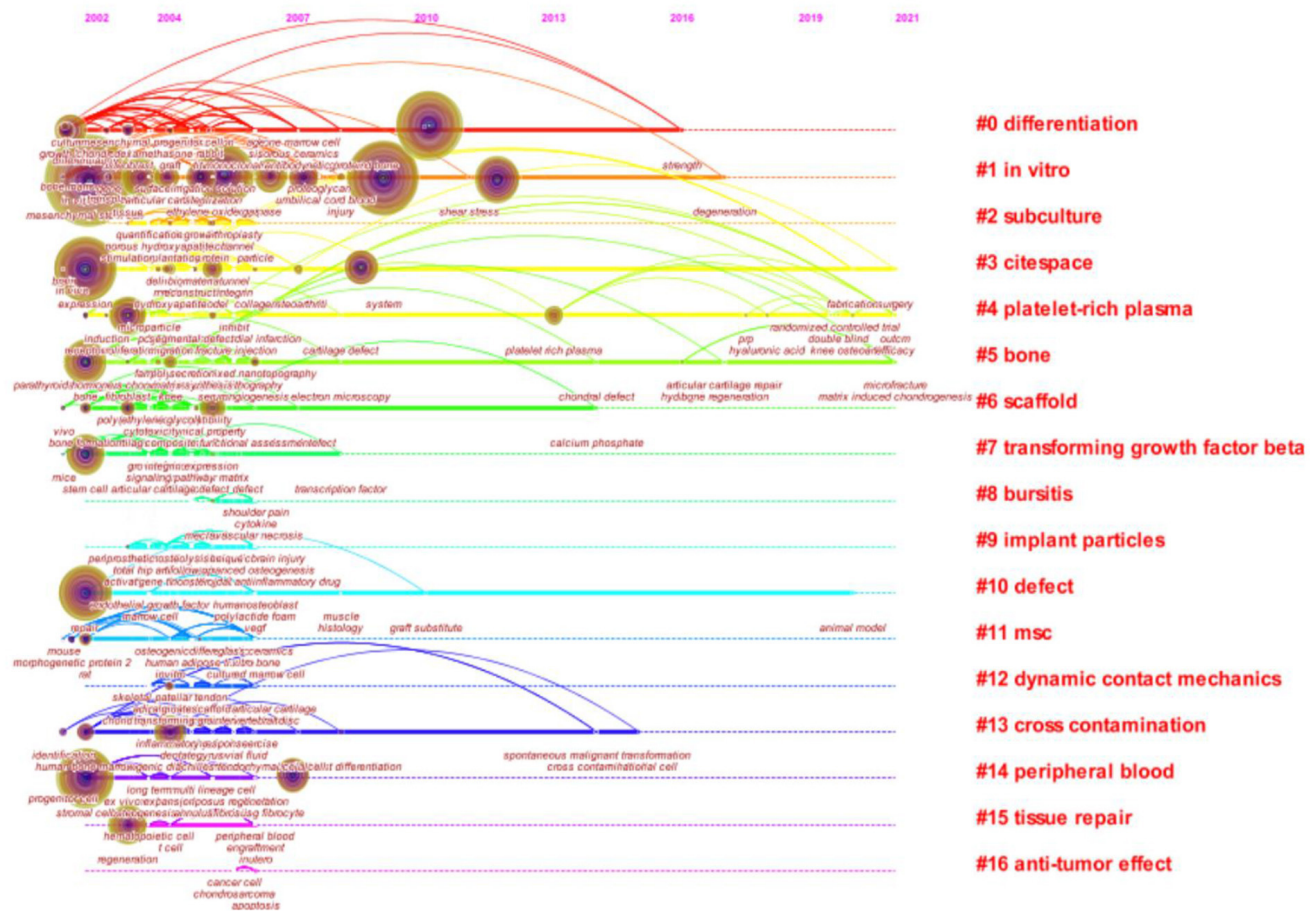
Keyword timeline visualization related to MSCs in orthopedics from 2002–2021. Abscissa x is the year of publication, and ordinate y is the cluster number. The more literatures the cluster contains, the more important the cluster is in the domain. The longer the time span, the earlier and longer the duration of the cluster.

**Figure 9:**
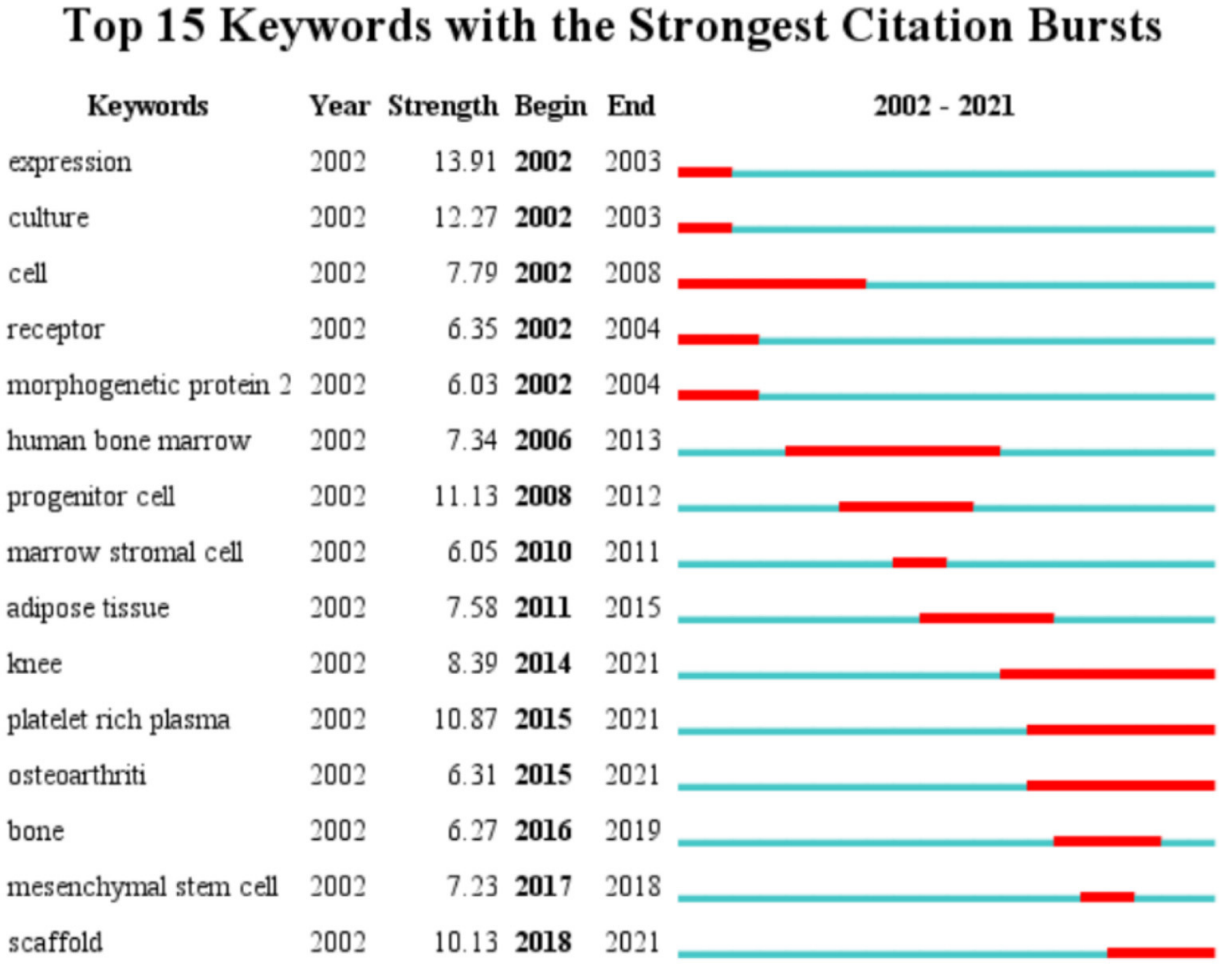
The top 15 keywords with the strongest citation bursts.

Keyword timeline visualization map mainly focuses on describing the relationship between clusters and the historical span of keywords in each cluster. Nodes in the same cluster were arranged on the same horizontal line in chronological order. Time was placed at the top of the view. The closer the node was to the right, the closer it was to the present time. The research history of MSCs in orthopedics can be clarified by the analysis of the keyword timeline visualization map. As can be seen from Figure 8, “*in vitro*,” “differentiation,” “platin-rich plasma,” “cross contamination” played an important role in the field, and their clustering time span was long, which showed that these clustering labels were the key research content and formed a stable research direction in the field. And “scaf-fold” and “bone” were another hot topic in recent researches. The keywords with strong bursts refer to the words that appeared frequently or were applied significantly more frequently in the short term, which can reflect the change and trend of the research direction in this field from a certain extent. The keywords were identified and analyzed using Citespace's strong citation bursts to explore the frontier field of MSCs in orthopedic diseases. As shown in Figure 9, the keywords with strongest citation bursts were “expression” (2002–2003), “culture” (2002–2003), and “progenitor cell” (2008–2012). The most recent burst keyword included “knee” (2014–2021), “platelet rich plasma” (2015–2021), “osteoarthritis” (2015–2021), and “scaf-fold” (2018–2021).

## Discussion

This study provided a bibliometric analysis of MSCs in orthopedics over the last 2 decades (2002 to 2021). As far as we know, this is the first bibliometric and visual analysis of MSCs in the field of orthopedics, which comprehensively reveals the research status and development trend in the field, and helps scholars analyze landmark research results and latest research hotspots in this field. We found that the research in this field reached a small peak in 2002, and then after a period of steady development, the number of publications in this field gradually climbed, and the average annual publications had been significantly increased compared with 20 years ago, up to 136. With the resumption of production after the COVID-19 pandemic and increasing fund investment, the amount of research in this field was still on the rise. This showed that scholars all over the world paid attention to the application of MSCs in orthopedic diseases, indicating that there is still a large potential for exploration in the future. The number of publications increased suddenly in 2002, which was considered to be the breakthrough of basic research on MSCs (18, 19). For example, in 2002, Bartholomew et al. (20) found that MSCs did not generate proliferation of allogeneic lymphocytes, and *in vivo* administration of MSCs could prolong the survival time of transplants. These immunomodulatory functions demonstrated in this study provided a strong theoretical basis for tissue regeneration and stem cell engineering in the future. Jiang et al. (21) showed that cells with the characteristics of multipotent adult progenitor cells could be separated not only from bone marrow but also from brain and muscle tissue. This study demonstrated that MSCs exist in multiple tissues, and their sources are extensive and convenient.

According to the visualization analysis of Citespace software, it can be seen that USA, China and Japan were the top three productive countries in research field of MSCs in orthopedic diseases. It was worth noting that USA published 553 articles from 2002 to 2021, accounting for 36.4% of the total number of publications, indicating that the country's scientific research strength was very strong. USA had more research institutes comparing with other countries. Among the top ten institutions with the number of publications, 5 of them belong to USA, indicating that USA is still the primary research area in this field. But at the same time, the number of publications in other countries was gradually increasing, and there were many high-quality articles. For example, Bourin et al. (22), from France, proved in 2013 that MSCs from fatty tissue could be distinguished from bone marrow by their positive for surface antigen CD36 and negative for CD106, which provided guidance for the scientific community to utilize MSCs derivied from fatty tissue and was widely recognized by scholars. In 2011, Davatchi et al. (23) from Iran injected MSCs extracted from bone marrow into knee joints with osteoarthritis at the first time, and the pain symptoms improved significantly.

The highly cited literatures reflected some important research achievements and indirectly proved that these fruits are the research basis of this field. Although the number of publications in China ranked the second (230), the total number of citations only ranked the fifth, indicating that there were still few basic studies published in this field in China, and efforts are still needed to improve high-quality articles. The articles with high centrality represent the key literatures in this field. Although the Netherlands was not in the top 5 countries in publication quantity and total citation times, the average citation time was the highest (73.15). Rombouts et al. (24) found in 2003 that the *in vitro* proliferation of bone marrow-derived MSCs significantly reduced their homing in femur and spleen, which provided a strong basis for the application of MSCs in the precision treatment of injured sites.

At the same time, this study found that many institutions began to invest in the exploration of MSCs in orthopedic diseases, and the publications number (196) of top 10 institutions only accounted for 12.9% of the total number of publications. Shanghai Jiao Tong University (33) was the institution that had published the most papers, which reflected its great influence on MSCs in orthopedics. Its frequent cooperation institutions included University of Bologna and Columbia University. Of the top 10 institutions in number of publications, five belonged to American. This reflected the high level of American medical and scientific capabilities. However, as shown in the cooperation network map of institutions (Figure 4), cooperation between institutions was relatively rare and not closely connected. For the long-term development of MSCs in the field of orthopedics, it is recommended to strengthen cooperation among institutions in the future.

Among the top 10 journals by number of publications, *Journal of Orthopedic Research* (IF: 3.493) had the most publications (402), reflecting the international influence of this journal in the field of MSCs application in orthopedics. There were 4 journals with IF > 4, and *Biomaterials* (IF: 12.497) had the highest IF. *Biomaterials*, as the top journal of materials science, ranked the second in terms of the number of publications, indicating that MSCs in orthopedic disease has a promising application in the field of biomaterials and tissue engineering (25, 26).

Rochy S. Tuan (National Institutes of Health, USA) was the most productive author in this field, with 13 publications and an average of 74.38 citations. He also had a high centrality and was one of the authors who cooperated most closely with others. Tuan et al. found that cells derived from bone trabecular fragments of adult have the potential to differentiate into a variety of mesenchymal lineages cells *in vitro* (27). At the same time, Tuan et al. demonstrated that traumatic muscle-derived MSCs exhibited similar phenotypes as bone marrow-derived MSCs and also had differentiation potential (28). It can differentiate into osteoblasts, adipocytes and chondrocytes (29). Therefore, MSCs extracted from the injured muscle tissue of orthopedic trauma patients who need debridement treatment can be considered for regenerative medicine (30). Rochy S. Tuan was an early participant in this field and had an important influence on later researches. As MSCs had become a hot topic in the treatment of diseases, both positive and negative reports have aroused great interest of orthopedic surgeons. As the author with the highest average citation frequency, Scott A. Rodeo (100.9) had made outstanding contributions to the basic scientific research and clinical application of MSCs. He highlighted the lack of consensus on optimal preparation, source, delivery method, and dosage of MSCs as a biologic therapy, and the need to pay attention to their safety and efficacy in clinical use (31, 32). Further clinical prospective randomized controlled trials are needed to better determine how, when, and where MSCs are used for optimal efficacy. In addition, a number of high-impact authors, such as Zhen et al. (33) (Johns Hopkins University, USA) demonstrated in 2013 that transforming growth factor β1 in subchondral MSCs is key to initiating osteoarthritis, providing a potential therapeutic option to treat this disease.

In this study, the top three keywords with the high frequency were “Mesenchymal stem cell” (649), “*in vitro*” (304) and “Differentiation” (295). As for the keyword “Mesenchymal stem cell” (MSC), it was necessary to explore its original source. It was first proposed in 1999 that MSC could differentiate into mesenchymal tissue lineage, that is, single stem cells could still retain their multilineage potential when expanded into colonies. With the development of research, the huge therapeutic potential of MSCs has attracted the intense attention of biomedical scholars. At the same time, the Mesenchymal and Tissue Stem Cell Committee of the International Society for Cellular Therapy proposed minimal criteria for defining human MSC (34). Firstly, MSC must be plastic-adherent under standard culture conditions. Secondly, MSC must express CD105, CD73 and CD90 markers and lack expression of CD45, CD34, CD14 or CD11b, CD79α or CD19 and HLA-DR surface molecules. Thirdly, MSC must differentiate into osteoblasts, adipocytes and chondroblasts *in vitro*. The above criteria make it possible to compare the results of different studies on MSCs on the basis of different isolation and expansion methods and cell characterization. At the same time, with the study on MSCs derived from peripheral blood, fat and cord blood, it was found that they not only have similar phenotype and differentiation potential with bone marrow-derived MSCs, but also have a wide range of sources and convenient sampling, and have fewer complications compared with bone marrow cell extraction (35, 36). The keyword “*in vitro*” ranked second in citation times. As an important bridge between basic theories and clinical trials, cell experiment has always been the most direct and necessary method to study the mechanism and efficacy of MSCs. However, due to different sources of MSCs, different preparation methods, individual differences and other factors, many conclusions about *in vitro* have been controversial. There are still few clinical researches on MSCs, so *in vitro* is still the primary method of current research. As the third most cited keyword, “differentiation” is an important part in inducing the transformation of MSCs to osteocyte and chondrocytes. Studies have shown that Wnt/β-catenin (37), BMP/Smad (38), MAPK (39), PI3K/AKT (40), TGF-β, Notch, NF-kB and other signaling pathways play important regulatory roles in the differentiation of MSCs into bone or cartilage (41). Its positive or negative regulatory effects alone or in combination have also aroused active exploration by scholars. It was believed that with the sustainable development of molecular biology and the continuous advancement of basic research, the cognition of the differentiation regulatory network of MSCs will be gradually clear, and then the process of precise intervention will be achieved.

Morphogenetic protein 2, as a functional protein, can stimulate DNA synthesis and cell replication and promote the directional differentiation of MSCs into osteoblasts (42). “Knee” was one of the keywords with a long bursts time. For cartilage injury, meniscus injury, cruciate ligament rupture and other knee diseases, when the condition is serious, can not heal by itself, and at the same time, the operation is more difficult. Therefore, MSCs, as the most promising seed cells in the field of regenerative medicine, have gradually been applied to diseases of knee joint (9). Saw et al. (43) conducted an *in vivo* experiment, which injected MSCs or hyaluronic acid into the articular cavity once a week for 5 weeks in patients with knee cartilage defects, and the results showed that the hyaline cartilage regenerated in the MSCs group, but not in the hyaluronic acid group. At the same time, MSCs can also differentiate into ligament fibroblasts, contributing to the regeneration of cruciate ligament, and the differentiation process can be regulated (44). In addition, it can also promote the repair of meniscus injury (45). Platelet-rich plasma (PRP) is a product of platelet concentration obtained from peripheral blood after repeated centrifugation, which has been used in clinical treatment since the 1980s (46). The application of PRP combined with MSCs in orthopedic diseases is another research hotspot, which has attracted the attention of many scholars. Ito et al. (47) demonstrated that tissue-engineered bone containing MSCs and PRP had better histological and mechanical properties than PRP-only graft material or autologous bone, so the combined protocol could be applied to early bone regeneration. In addition, studies on PRP also showed that the combined application of MSCs and PRP could significantly promote the maturation and repair of allogeneic tendon and ligament tissues (48). And “Scaf-fold”, as an emerging bursts keyword, is another research frontier now. Because of the rapid development of tissue engineering technology in recent years, the experimental research on the combination of seed cells and various scaf-folds has become a hot spot and is becoming increasingly mature. These biological scaf-folds have good biocompatibility and biodegradability, which are composed of inorganic materials (such as hydroxyapatite, tricalcium phosphate, bioactive glass), natural scaf-fold materials (such as collagen, fibrin, elastin, gelatin, hyaluronic acid, chitosan), synthetic polymers (such as polylactic acid, polycaprolactone, polyethylene glycol, polylactic acid-glycolic acid), titanium and other scaf-fold materials. These scaf-folds need to have sufficient mechanical strength and plasticity (49). The ideal scaffold can promote the osteogenic or chondrogenic differentiation of MSCs, facilitate the arrangement of tension fibers, cell expansion and differentiation, and also play a crucial role in regulating the adhesion and proliferation of MSCs (50). For example, Wang et al. (51) prepared a double-layer bionic cartilage scaf-fold by simulating the structure, chemical and mechanical properties of mature articular cartilage. The surface layer of the scaf-fold was made of collagen, chitosan and sodium hyaluronate, and the transition layer with microtubule array structure was prepared with collagen, chitosan and silk fibroin. Then, the MSCs-loaded scaf-fold was implanted into a rabbit model of knee osteoarthritis cartilage defect. The results showed that the bionic cartilage scaf-fold could induce the proliferation and differentiation of MSCs and effectively repair the cartilage defect. It is worth noting that the lack of vascularization in addition to osteogenic induction is the next problem for stem cells to be solved by biological scaf-folds (52), Growth factors play an irreplaceable role in the regulation of angiogenesis and osteogenic differentiation. Therefore, future bone tissue engineering will focus on the composite of biological scaf-folds through different manufacturing technologies and the addition of growth factors and other chemotactic peptides to maximize the function of MSCs. However, many studies were still in the stage of *in vitro* or animal experiments. The author believes that with the in-depth research, the application of MSCs in orthopedic diseases will make further breakthrough progress.

This is the first time in the orthopedic field to perform bibliometric and visual analysis on mesenchymal stem cells. Despite the fact that rigorous and standard bibliometric analysis have been performed, there are still some unavoidable limitations. Firstly, we only retrieved publication from the WoS database, which may have missed relevant publications reported in other databases, such as Scope and PubMed (53). In addition, we only recruit publications in the English language, which may cause some important non-English research in this area to be overlooked. Finally, we did not include the most recent publications in 2022 because they lacked sufficient time to accumulate a large number of citations, which may influence our conclusions about the research frontier to some extent.

## Conclusion

In general, this study summarized the valuable information and research trends in the field of MSCs in orthopedics by bibliometric analysis. The USA, China, Japan and the United Kingdom were the primary contributors in this field. Shanghai Jiao Tong University was the most productive institution. *Journal of Orthopedic Research* was the journal that published the most Research results. *Experimental Hematology* was the journal with the highest average citation frequency. Rochy S. Tuan was the most productive author. Scott A. Rodeo was the most cited author on average. While there was not much collaborations among the authors in this field, showing that increased collaboration is needed to boost the development of MSCs research in orthopedics. The main research hotspots of MSCs in orthopedics are the source of MSCs, *in vitro* experiments, and the differentiation of MSCs into bone and chondrocytes. The frontiers of this field are the combination of MSCs and PRP, the treatment of knee diseases, osteogenic differentiation, and the application of biological scaf-folds combined with MSCs. Meantime, it can be seen that the research focus of MSCs has gradually changed from the basic research level of cell culture to clinical application. However, clinical transformation of MSCs requires clinical trials with larger sample size and longer follow-up period. All in all, we believe that this bibliometric study can provides a new perspective for researchers to quickly acquire the background knowledge and research hotspots in this field. It also helps exploring new research directions, such as the combined application of MSCs with other biological agents, or endophytic plants, the potential of MSCs in tissue engineering, and the innovation of special MSCs dosage forms for different orthopedics diseases.

## Data availability statement

The original contributions presented in the study are included in the article/supplementary material, further inquiries can be directed to the corresponding author.

## Author contributions

All named authors have substantially contributed to conducting this research and drafting this manuscript. ZD and FL designed the experiments and wrote this manuscript. YL and JL searched articles and extracted data. DK and CS made the visualized analysis. JX examined the original study data, provided technical guidance, and approved the final manuscript. All authors read and approved the final manuscript.

## Funding

This work was supported by the Joint key projects of Fujian Province in 2019 (2019-WJ-01), the Medical Innovation Project of Fujian Provincial Health Department (2019-CX-1), the Firestone Research Project of Fujian Provincial Hospital (2020029HSJJ), the Fujian Provincial Natural Science Foundation Projects (2020J05270), the Medical Innovation Project of Fujian Provincial Health Department (2020QNA009), the Major Scientific Research Project of Fujian Province (2021ZD01003), and the Fujian Provincial Natural Science Foundation Projects (2021J01376).

## Conflict of interest

The authors declare that the research was conducted in the absence of any commercial or financial relationships that could be construed as a potential conflict of interest.

## Publisher's note

All claims expressed in this article are solely those of the authors and do not necessarily represent those of their affiliated organizations, or those of the publisher, the editors and the reviewers. Any product that may be evaluated in this article, or claim that may be made by its manufacturer, is not guaranteed or endorsed by the publisher.
